# Non-covalent dyes in microscale thermophoresis for studying RNA ligand interactions and modifications†

**DOI:** 10.1039/d3sc02993j

**Published:** 2023-08-29

**Authors:** Elisabeth Kallert, Malte Behrendt, Ariane Frey, Christian Kersten, Fabian Barthels

**Affiliations:** a Institute of Pharmaceutical and Biomedical Sciences, Johannes Gutenberg-University of Mainz Staudingerweg 5 55128 Mainz Germany barthels@uni-mainz.de

## Abstract

Microscale Thermophoresis (MST) is a powerful biophysical technique that measures the mobility of biomolecules in response to a temperature gradient, making it useful for investigating the interactions between biological molecules. This study presents a novel methodology for studying RNA-containing samples using non-covalent nucleic acid-sensitive dyes in MST. This “mix-and-measure” protocol uses non-covalent dyes, such as those from the Syto or Sybr series, which lead to the statistical binding of one fluorophore per RNA oligo showing key advantages over traditional covalent labelling approaches. This new approach has been successfully used to study the binding of ligands to RNA molecules (*e.g.*, SAM- and PreQ_1_ riboswitches) and the identification of modifications (*e.g.*, m^6^A) in short RNA oligos which can be written by the RNA methyltransferase METTL3/14.

## Abstract 2

Time to shine a light on RNA: this protocol presents an innovative technique to study RNA using nucleic acid-sensitive dyes in MST.

## Graphical abstract

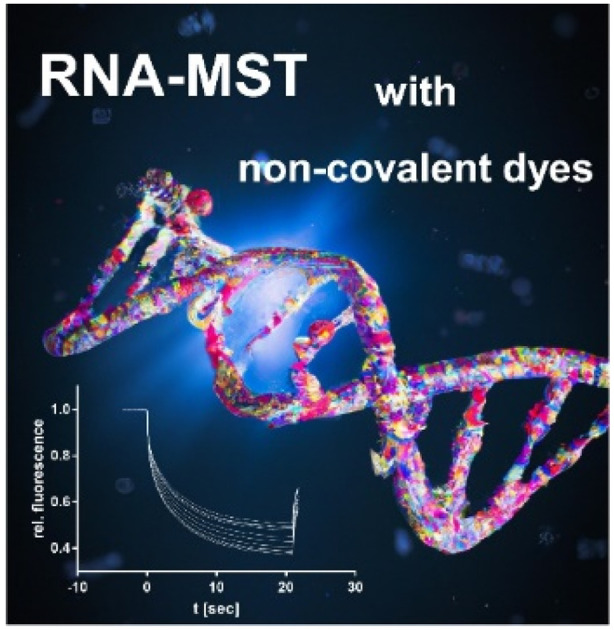

## Introduction

Understanding the molecular foundations of RNA biomolecular interactions is crucial for the study of their cellular involvement and the development of RNA-targeting therapeutics.^1,2^ Microscale thermophoresis (MST) is a powerful biophysical technique for the study of the interactions between molecules such as proteins or nucleic acids with high sensitivity and specificity.^3,4^ MST measures their mobility in response to a temperature gradient (usually 2–5 °C), and thus, the measured thermophoresis is dependent on the inherent properties of the biomolecule, such as its size, shape, and charge, while the origin is governed by the Soret coefficient.^5^ In recent years, MST has been utilized in the study of RNA molecules, providing valuable insights into the dynamics and interactions of these intensively researched biomolecules.^6–8^ The target RNA is commonly labelled with a covalent fluorescent reporter; subsequently, the sample is placed in a microliter-scale capillary, and a temperature gradient is applied by heating with an IR laser. Traditional MST methods for RNA studies rely on reporter dyes such as Cy5 or fluorescein, which are synthetically attached to the 3′- resp. 5′-ends, or to modified nucleosides.^6,8–11^ While this is effective to prevent steric interaction of the fluorophore with the investigated biomolecular interface, it might also reduce the effect of local structural changes (T-jump effects) on the dyes' fluorescence.^12,13^

In this study, we present a novel MST-based methodology for the investigation of RNA-containing samples using nucleic acid-sensitive dyes, such as those from the Syto or Sybr series, which statistically bind to nucleic acids through non-covalent interaction.^14^ It is important to note that most of these dyes do not significantly alter overall RNA stability which is a prerequisite for the study of biomolecular interactions.^15^ To our knowledge, there is only one published study which has used non-covalent SybrGold labelling of DNA oligos using a non-microscale thermophoresis setup (microscope detection) to show that Sybr-DNA duplexes do not dissociate under common thermophoretic conditions.^16^ In this previous study, however, no relation was made to applications in the recently popular MST experimental set-up nor to method development for nucleic acid analysis.

**Fig. 1:**
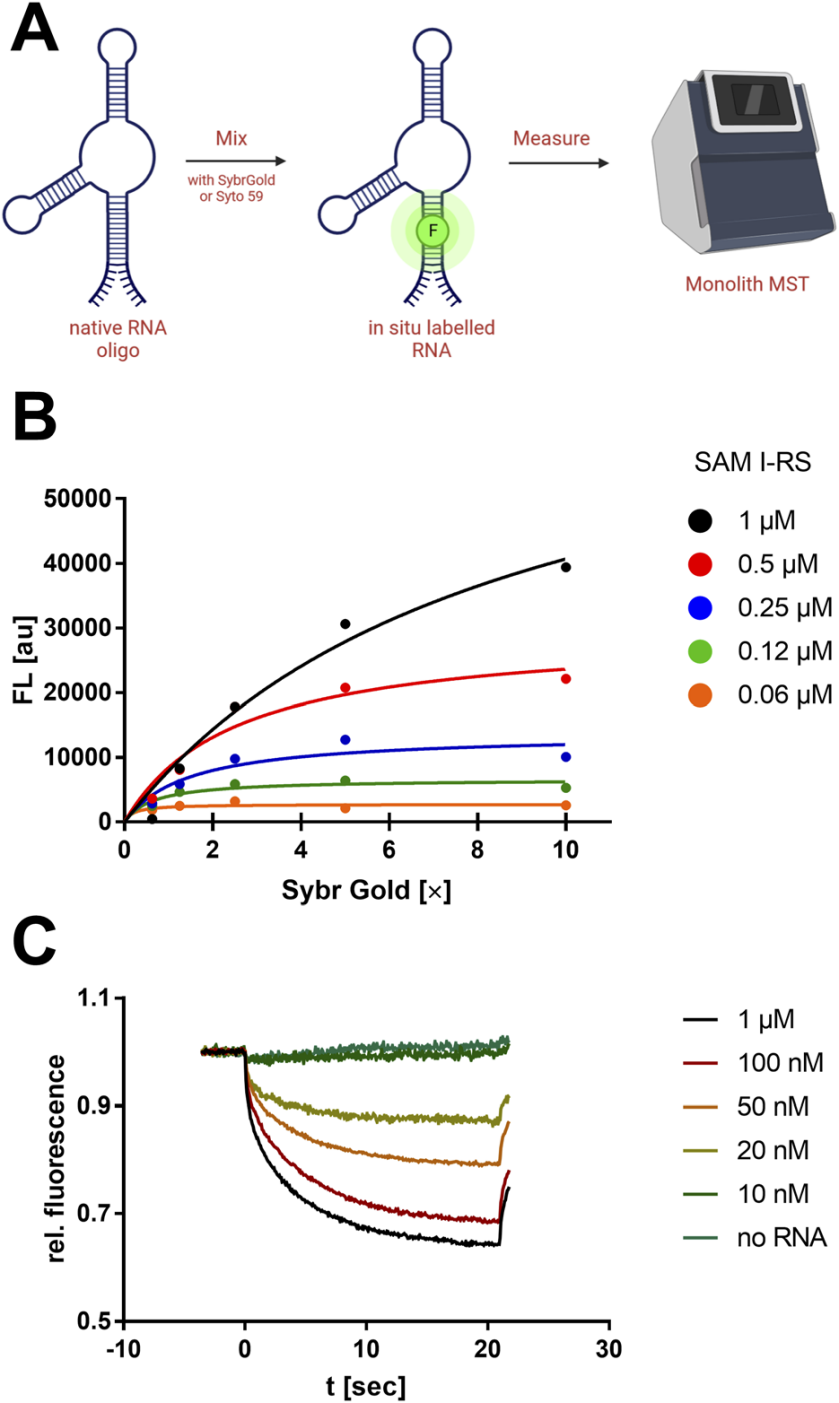
Non-covalent nucleic acid dyes to study RNA by MST. (A) “Mix-and-measure” protocol for the thermophoretic analysis of native RNA oligos. (B) Fluorometric titration of SAM-I riboswitch with SybrGold. Samples were measured by a Tecan Spark 10 M plate reader (*λ*_ex/em_: 490/550 nm). The apparent binding constant (Langmuir isotherm) was determined to be *K*_D_ = 85.3 nM. (C) RNA concentration-dependent MST curves with SybrGold (1×) and SAM-I riboswitch (variable concentration, blue laser, 8% excitation power). The apparent *K*_D_ from the *F*_norm_ (2 s) was 61.4 nM.

This led us to hypothesize that such nucleic acid dyes are also suitable for RNA labelling in MST experiments, and hence, we elaborated on the experimental scope, structure–activity relationships and practical applications for RNA–ligand and RNA-modification elucidation. Here, we report a “mix-and-measure” protocol that shows key advantages for relevant RNA-MST applications compared to covalent labelling approaches which are technically more challenging, time-consuming, and expensive (Fig. 1A). This approach thus pays homage to the dye-nitrilotriacetic acid (NTA) protocol developed for protein MST, which allows non-covalent labelling of proteins and has subsequently become more widely used than the original covalent maleimide- and NHS-labelling protocols.^17^

## Results and discussion

### Establishment of the non-covalent dye MST protocol

First, a qualitative working range of useful SybrGold and RNA concentrations was determined by fluorometry. With a sample matrix of RNA (62.5 nM–1 μM) and SybrGold (1×–10×) concentrations, the stoichiometry and apparent binding constant of the dye to the *S*-adenosylmethionine (SAM)-I riboswitch was determined (Fig. 1B).

It was found that 1× SybrGold produced sufficient fluorescence for MST (Monolith NT.115), and at such SybrGold concentrations, one fluorophore molecule is likely to bind statistically to the RNA of interest (*K*_D_ = 85.3 nM). Statistical 1 : 1 binding stoichiometry was concluded as a one-step binding event was observed to display the same apparent binding affinity independent of the RNA concentration. Hence, it was found that the minimum RNA concentration during MST was primarily limited by the binding affinity of SybrGold to the respective RNA sequence. For the SAM-I riboswitch, a significant thermophoretic response was saturated at an RNA concentration of approx. 100 nM, which was strongly distinguished from the unbound dye. *F*_norm_ (2 s): 0.75 *vs.* 0.96 (Fig. 1C).

**Fig. 2:**
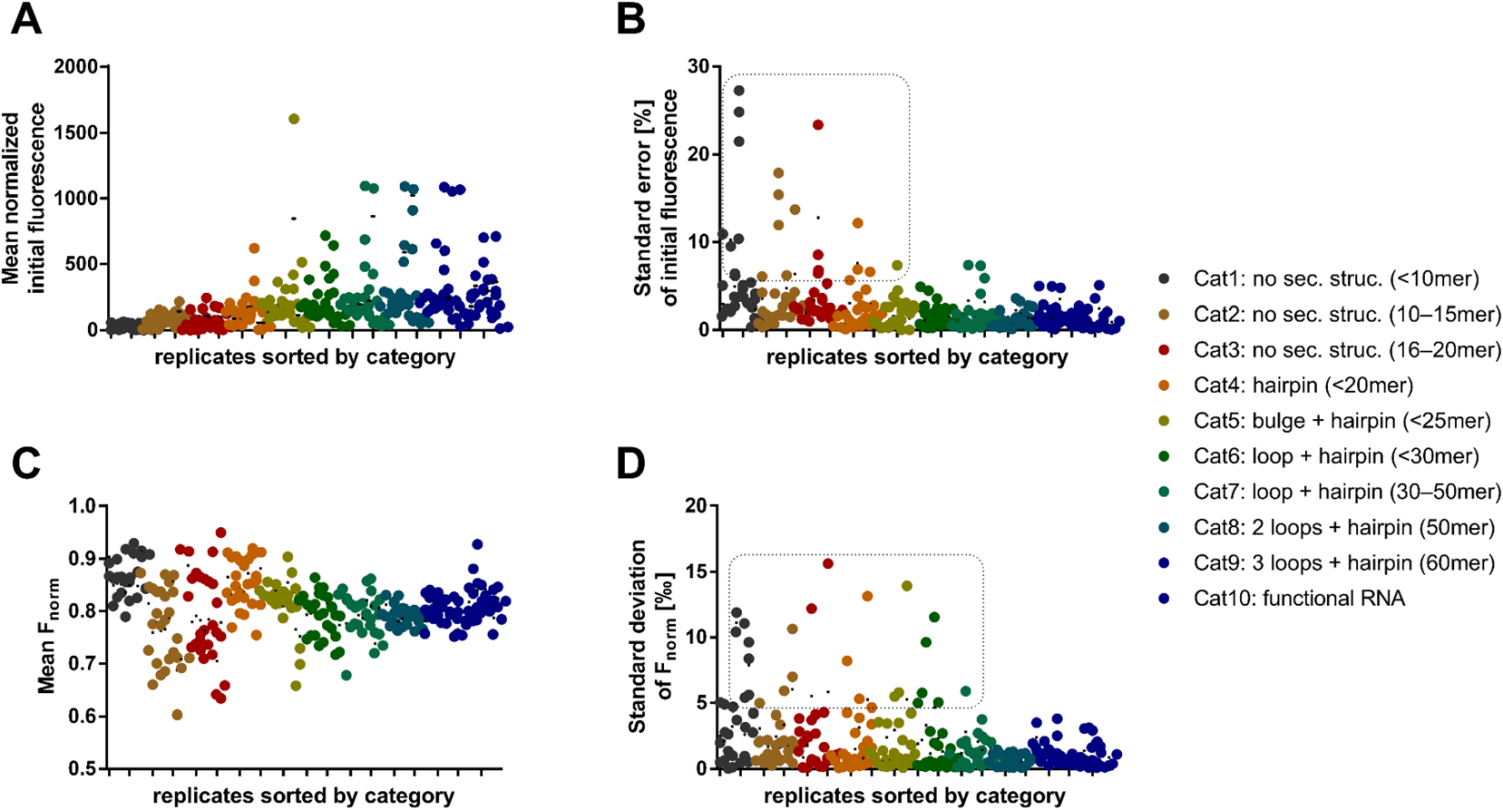
Initial fluorescence and *F*_norm_ as RNA-dependent MST characteristics. (A) Mean normalized initial fluorescence of all dye combinations for each RNA oligo. (B) Relative standard error of the mean normalized initial fluorescence. For Syto 62 combinations with short RNAs (category 1–5) the error is >5% (dashed box). (C) The key MST descriptor *F*_norm_ (*t* = 2 s) displays mostly values between 0.75 and 0.90 across all categories and dyes. (D) Standard deviation of the *F*_norm_ values (*t* = 2 s).

As a next step, systematic investigations of structure–activity-relationships between fluorescent dyes and RNA in MST were performed. First, we investigated the effect of RNA length and its secondary structures on the MST characteristics, such as the initial fluorescence level and *F*_norm_, using nine different dyes (Syto 17, Syto 59–64, SybrGold, and RiboGreen). For this purpose, we defined ten RNA categories containing each three RNA oligos which comprise different lengths and structural features (Table S1†). Across all dye and RNA combinations, MST mixtures were prepared as triplicates with 100 nM RNA and 1× dye. The mean initial fluorescence of different dyes was normalized by a concentration-dependent regression (Fig. S1†). In general, more complex RNA structures and longer oligonucleotide sequences resulted in higher absolute initial fluorescence (Fig. 2A). The central MST descriptor *F*_norm_ (2 s) was mostly between 0.75 and 0.90 across all combinations (Fig. 2C). Lower *F*_norm_ values (0.60–0.75) were eventually found for shorter RNA, however, a significant dependency on the dye or the category was not ascertained.

We found that shorter RNAs (<20 nt) exhibit more sensitive fluctuations in *F*_norm_, suggesting that these are more susceptible to biological and chemical modifications (Fig. 2C). This can possibly be exploited to detect small changes in sequence or conditions for biological assay design. Depending on the dye, however, the standard error of the absolute fluorescence and *F*_norm_ was coincidently higher for these short RNAs. The standard deviation of the *F*_norm_ values (*t* = 2 s) was mostly <5‰ across all categories. An exception was the use of the dyes Syto 62 and RiboGreen. The data points in the dashed box of Fig. 2D are exclusively populated by RNA–dye combinations with Syto 62 or RiboGreen. For Syto 62, this may be due to the highly variable absolute fluorescence of the RNA–dye conjugates (Fig. 2B). The use of these two dyes might be not recommended for RNA-MST experiments.

After the broad analysis regarding the influence of RNA-dependent parameters on the initial fluorescence and *F*_norm_, more detailed correlation studies with RNA descriptors (length, secondary structure, and base composition) were performed. The secondary structures of RNA oligos were predicted by the RNAfold web server.^18^ By analyzing the relationship between *F*_norm_ and RNA length, we confirmed the trend shown in Fig. 2, wherein shorter RNAs (<20 nt) lead to a greater diversity of *F*_norm_ (Fig. S2A†). Notably, the *F*_norm_ parameters of the two blue dyes (SybrGold and RiboGreen) were generally lower than that of the red Syto dyes. High standard deviations (>5‰) were found only for Syto 62 and RiboGreen. As further correlation parameters, the proportion of hybridized bases (Fig. S2C†) and the secondary structural content were investigated (Fig. S2E†). Neither the analysis of the paired bases nor that of the loop/bulge content *vs. F*_norm_ yielded a significant correlation between the given quantities (Fig. S3†).

**Fig. 3:**
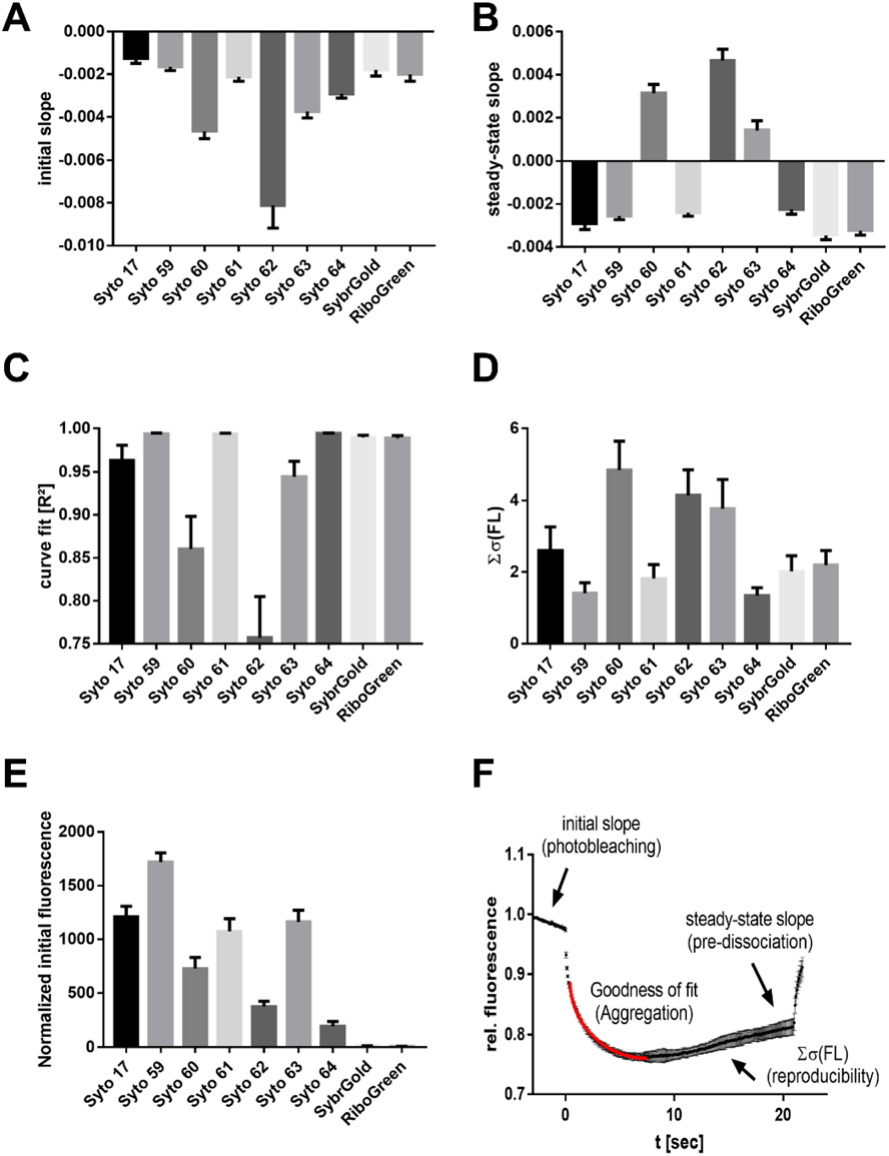
Dye-dependent effects on the MST characteristics. (A) Initial slope indicating the photobleaching rate. (B) Steady-state (*t* = 15–20 s) slope showing the stability of dye–RNA complexes. (C) Goodness of the curve fit (*R*^2^). (D) Dye-dependent reproducibility. (E) Normalized initial fluorescence. (F) Depiction of the MST curve descriptors analyzed in subfigures (A)–(D).

Another aspect of structure–activity relationships was the analysis of dye-dependent parameters on characteristics of thermophoresis curves described by the following descriptors (Fig. 3F): (1) slope of the pre-MST fluorescence traces (measure of dye photobleaching), (2) steady-state (*t* = 15–20 s) slope of thermophoresis traces (measure of the RNA–dye complex dissociation in the temperature gradient), (3) goodness (*R*^2^) of the hyperbolic thermophoresis fit, (4) sum of the least squares error of a fluorescence–time curve triplicate (reproducibility of the whole MST experiment), (5) normalized initial fluorescence (measure for the brightness of the intrinsic dye fluorescence).

These results showed that Syto 60, 62, 63, and 64 exhibited increased susceptibility to photobleaching, with Syto 62 showing a particularly strong effect (Fig. 3A). In contrast, Syto 17, 59, and 61 as well as SybrGold and RiboGreen were mostly resistant to photobleaching. Furthermore, the dye–RNA complex of Syto 60, 62, and 63 seem to be prone to premature dissociation during thermophoresis, as a positive steady-state slope was observed for these dye combinations (Fig. 3B). We also found varying degrees of susceptibility to aggregation for Syto 17, 60, 62, and 63 during thermophoresis, while the other dyes were stable across all types of RNA samples. Regarding the reproducibility of an MST experiment triplicate, Syto 62 as well as Syto 60 and 63 led to significantly lower reproducibility compared to the other dyes (Fig. 3D). Lastly, Syto 59 is by far the brightest in terms of its intrinsic fluorescence compared to the other red dyes (Fig. 3E).

To summarize, we found that Syto 59 (red, pico channel) and SybrGold (blue, nano channel) are the most suitable (of the investigated) dyes for RNA-MST experiments, as they showed the best fluorescence benchmarks and highest reproducibility rates.

### Investigation of RNA ligand binding

**Fig. 4:**
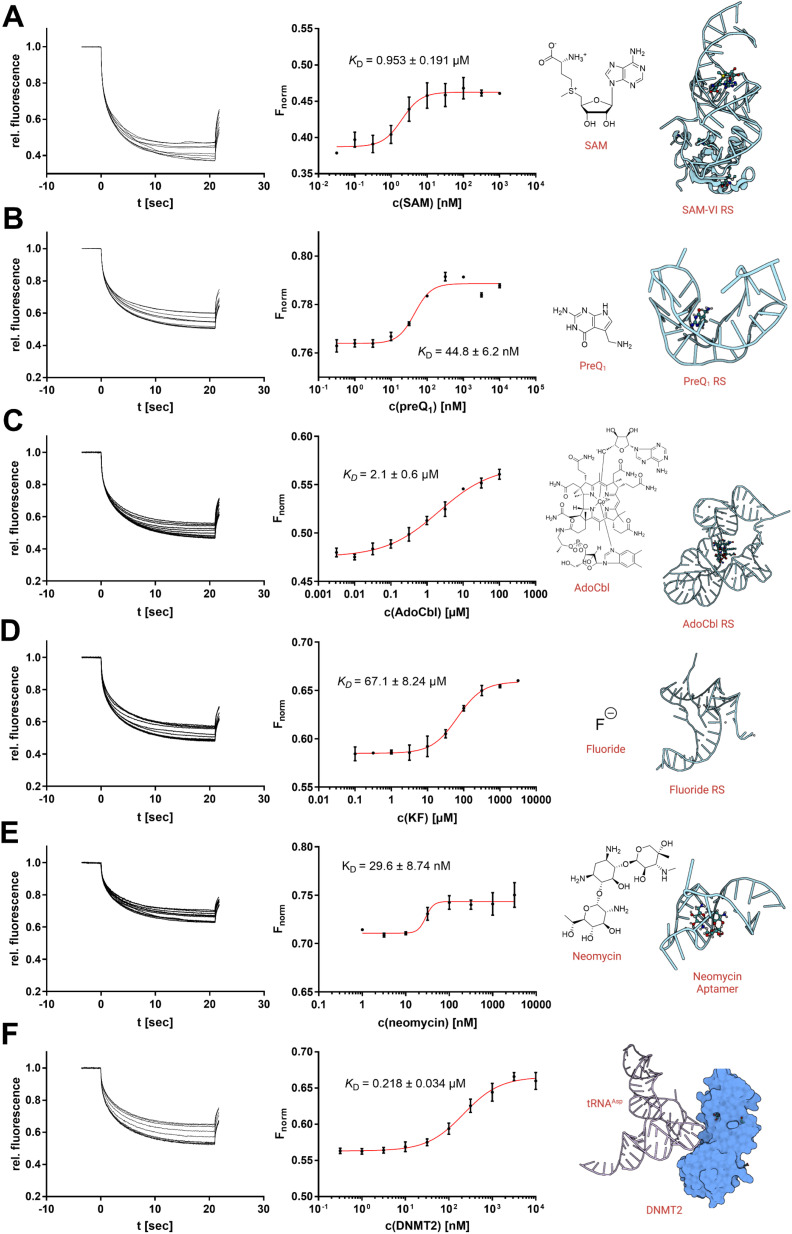
Analysis of RNA–ligand binding by *in situ* labelling with SybrGold or Syto 59 (1×). Dissociation constants (*K*_D_) were determined using a Boltzmann fit. (A) Interaction between SAM-VI RS and SAM (*F*_norm_ at 20 s, SybrGold). (B) PreQ_1_ RS and the PreQ_1_ ligand (*F*_norm_ at 1 s, SybrGold). (C) AdoCbl RS and the AdoCbl ligand (*F*_norm_ at 15 s, Syto 59). (D) Fluoride RS and the potassium fluoride ligand (*F*_norm_ at 5 s, Syto 59). (E) Neomycin aptamer and the neomycin ligand (*F*_norm_ at 5 s, Syto 59). (F) tRNA^Asp^ and DNMT2 (*F*_norm_ at 10 s, SybrGold).

Targeting physiologically relevant RNA with small molecule drugs has become of increasing interest as a potential drug target over the last years.^1,19,20^ Currently, the standard repertoire of bioanalytical techniques includes techniques such as nuclear magnetic resonance (NMR), surface plasmon resonance (SPR), and isothermal titration calorimetry (ITC).^21,22^ However, in recent years, MST has probably become one of the most popular techniques for the characterization of RNA-small molecule interactions using covalently dye-labelled RNA targets.^6,8,10^ Hence, we aimed to demonstrate four examples where the use of SybrGold and Syto 59 (Fig. 4 and S4†) showed comparable results to RNA ligand binding profiles as determined with orthogonal techniques.

Thus, we investigated three types of nucleotide-derived ligand riboswitch (RS) aptamer domains: the *T. tengcongensis* SAM-I RS, the *B. angulatum* SAM-VI RS, and the *T. tengcongensis* pre-queuosine 1 (preQ_1_) RS, with their natural ligands SAM, *S*-adenosylhomocysteine (SAH), and preQ_1_, respectively.^23–25^ For the SAM-I RS, our MST data yielded a *K*_D_ = 157 nM for SAM and *K*_D_ = 137 μM for SAH agreeing with previously reported values obtained by ITC measurements.^26^ For the SAM-VI RS, our MST results for SAM (*K*_D_ = 953 nM) and SAH (*K*_D_ = 10 μM) were also consistent with our covalently Cy5-labelled MST experiments (Fig. S5†) and literature reports.^27,28^ Similarly, our results showed that preQ_1_ binds the preQ_1_ RS with an apparent *K*_D,app_ of 44.8 nM.

While this value is in the same order of magnitude as our previously reported Cy5-labelled RNA affinity (Fig. S5†), it should be noted that high-affinity ligands (<50 nM) will result in tight-binding to the RNA receptor ([RNA] = 100 nM) and thus imply some scope limitation of this presented protocol.^6,8^ For the existing protocols using a covalently labelled RNA target, however, this mostly represents the same technical limitation.

As a next step, we investigated three additional RS aptamer domains against chemically more diverse chemotypes of ligands: a marine metagenome AdoCbl RS, the *T. petrophila* fluoride anion RS, and a synthetic neomycin aptamer.^29–31^ The AdoCbl RS naturally binds different variants of the cobalamin co-factor family, which are exceptionally large ligands with a molecular weight of >1000 Da. Our MST experiments using an env8 mutant AdoCbl RS with Syto 59 labelling determined a *K*_D_ value of 2.1 μM with adenosylcobalamin (AdoCbl) as a ligand, which is in agreement with the literature.^29^ Thus, the non-covalent RNA-MST method is also suitable for non-typically large ligands and their riboswitches.

Next, we studied the opposite case; a riboswitch that is able to bind very small ligands. Aptamers against the smallest known ligands are fluoride anion aptamers. The study of the *T. petrophila* fluoride anion RS showed that this protocol is also useful for such applications if ligand binding results in a substantial conformational change of the aptamer. Hence, non-covalent RNA-MST experiments with Syto 59 (*K*_D_ = 67.1 μM) are in the same order as dissociation constants determined by ITC experiments in the literature.^30^

As a final example, we examined a neomycin-binding aptamer of synthetic origin. The apparent binding affinity of neomycin to the 27-mer RNA oligo could be determined by *K*_D,app_ of 29.6 nM.^31^

Of note, the titration of *in situ* labelled RNA samples with their natural ligands mostly does not lead to a change in the fluorescence intensity by displacement of the dye with the ligand (see MST capillary scans in Fig. S8†). In this regard, an RNA target usually has just one ligand binding resp. interaction site but possibly several dye binding sites, some of which overlap with the ligand binding site and some of which do not. If we choose the concentrations of RNA (100 nM) and dyes (1×) such that statistically at most 1 dye molecule is bound per RNA (Fig. 1B), there will be functional, fluorescent RNA molecules in solution that we can (a) address with the ligand resp. interaction and (b) measure their altered thermophoresis behaviour. For the very short PreQ_1_ riboswitch or the neomycin aptamer (both 27 nt), however, we quantified a slight effect on the initial fluorescence at higher ligand concentrations which supports our model of statistical labelling, since for such a short RNA receptor there are fewer opportunities for dye binding that do not overlap with the ligand binding site (Fig. S8A&G†).

Furthermore, we also investigated a macromolecular interaction of RNA with an endogenous protein–ligand. The tRNA^Asp^ represents the physiological substrate of the RNA methyltransferase DNMT2.^32^ Using SybrGold, we were able to determine a dissociation constant of this macromolecular interaction (*K*_D_ = 0.218 μM) similar to a value determined by fluorescence polarization.^33^

### Investigation of RNA nucleobase modifications

The development of methods for the detection and quantification of RNA modifications such as nucleobase methylation is a recent topic of research due to their physiological relevance and disease involvement.^34^ Currently, the gold standard repertoire of bioanalytical techniques includes mass spectrometric, radiometric, and sequencing methods.^35^ Due to the simple and fast preparation of an MST experiment, the development of an MST protocol for the quantification of RNA modifications might offer practical advantages. Thus, the two most promising fluorescent dyes (SybrGold & Syto 59) were harnessed to test for the detection of specific RNA nucleoside modifications.

**Fig. 5:**
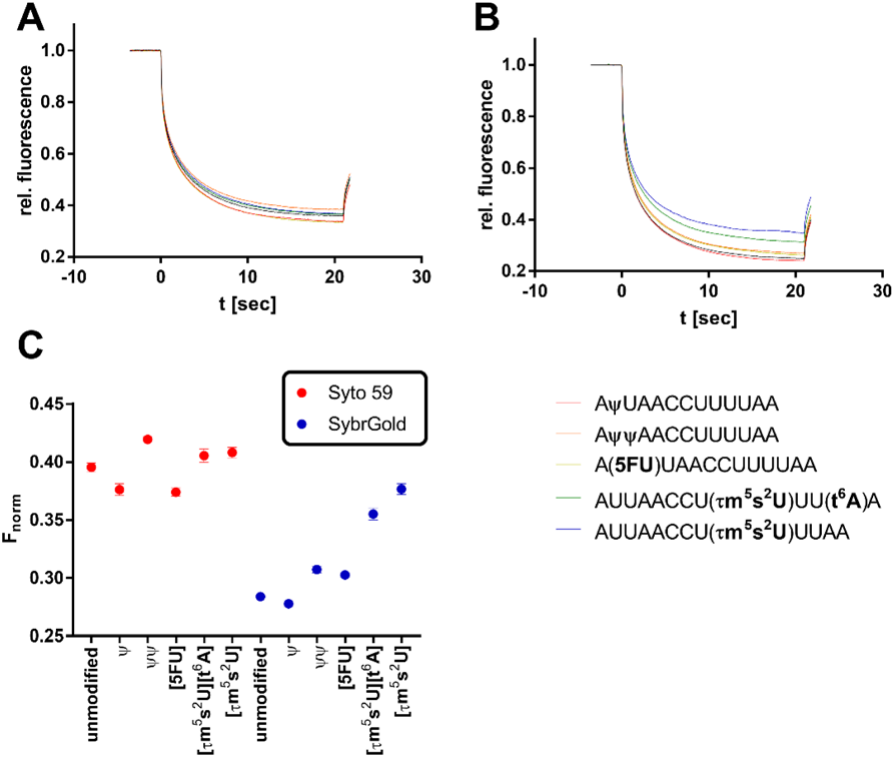
Analysis of various modifications of the model oligo AUUAACCUUUAA (100 nM) by MST. (A) Thermophoresis curves for the usage of Syto 59 as a reporter dye. (B) Thermophoresis curves for the usage of SybrGold as a reporter dye. (C) MST response (*F*_norm_ at 10 s) of the modified oligos in comparison to their unmodified analogues.

**Fig. 6:**
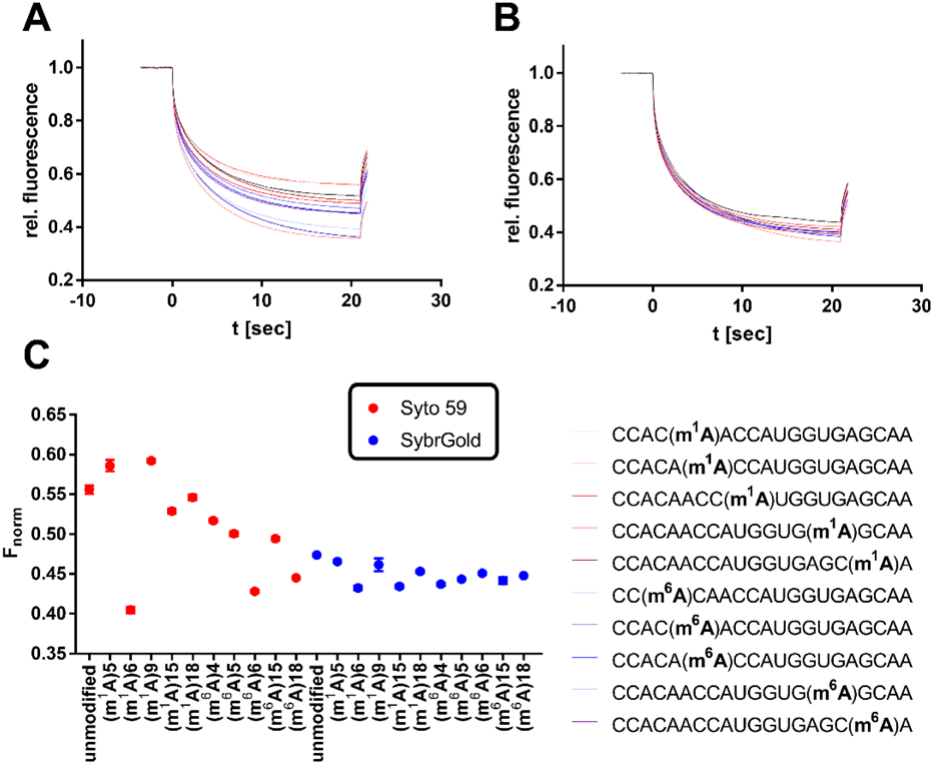
Analysis of various modifications of the model oligo CCACAACCAUGGUGAGCAA (100 nM) by MST. (A) Thermophoresis curves for the usage of Syto 59 as a reporter dye. (B) Thermophoresis curves for the usage of SybrGold as a reporter dye. (C) MST response (*F*_norm_ at 10 s) of the modified oligos in comparison to their unmodified analogues.

First, as a proof of concept, two variously modified model RNA oligos AUUAACCUUUUAA (Fig. 5) and CCACAACCAUGGUGAGCAA (Fig. 6 and S6†) were investigated for changes in their *F*_norm_ parameters in dependence on specific RNA modifications. In summary, these model experiments showed that larger or charge-altering RNA modifications (*e.g.*, 5-taurinomethyl-2-thiourididylation) have a more pronounced effect on *F*_norm_ than single nucleoside methylations (*e.g.*, 6-methyladenine, or 2′-*O*-methylation), which often resulted in scarcely detectable *F*_norm_ shifts (Fig. 5, 6, and S6†).

In the first step, the influence of different physiologically relevant modifications such as pseudouridylation (ψ), 5-uridine fluorination (5FU), 5-taurinomethyl-2-thiourididylation (τm^5^s^2^U), and threonylcarbamoyladenosylation (t^6^A) on the short non-structured model oligonucleotide AUUAACCUUAA as a fragment of the hmt-tRNA^Lys^ was investigated.^36^

Significant divergences were found between the two dyes SybrGold and Syto 59 in the differential analysis of the *F*_norm_ values. While Syto 59 showed only slight changes in *F*_norm_ values depending on the modifications (Fig. 5A), significant increases in *F*_norm_ values were measurable when SybrGold was used, especially for the two oligos with the 5-taurinomethyl-2-thiourididylation (Fig. 5B). A more systematic investigation of a second model oligonucleotide (CCACAACCAUGGUGAGCAA) was conducted. This is a generically designed oligo that has a hairpin with three regions (stem, loop, tail). The hypothesis was to show at which position RNA methylations have a particular effect on the MST shift For this purpose, the adenine positions of the unmodified parent oligo were systematically methylated either at the N^1^ or N^6^ position of an adenine base (Fig. 6), or the 2′-OH group of ribose was methylated (Fig. S6†).

In contrast to the results shown in Fig. 5, the detection of adenine methylation with Syto 59 led to more sensitive changes in *F*_norm_ values than with SybrGold (Fig. 6C). In particular, m^1^A or m^6^A methylation of the 6th adenine base resulted in significantly altered *F*_norm_ values compared to the unmodified oligo indicating that local influences of RNA modification either affect the binding of the fluorophore or these modifications modulate the global topology resp. folding of the RNA and thus lead to an altered thermophoresis behaviour. The detection of ribose methylation followed a similar trend, however, with this form of modification leading to generally lower changes in *F*_norm_ values (Fig. S6C†).

### Development of an MST-based METTL3/14 enzyme assay

To derive a relevant biotechnological application from this model concept, an MST-based enzyme assay was developed for the RNA methyltransferase METTL3/14 allowing the tracking of the enzymatic insertion of a single methyl group to an RNA substrate. METTL3/14, which has recently become popular as a potential drug target, is an RNA methyltransferase that transfers a methyl group from SAM to the N^6^ atom of an adenine base found in a GGACU motif.^37^ Thus, we focused on the development of an enzymatic assay that can follow the 6-methyladenine (m^6^A) methylation of the METTL3/14 substrate oligo AACUUAAUGUUUGCAUUGG(m^6^A)CUUGAGUUA.

**Fig. 7:**
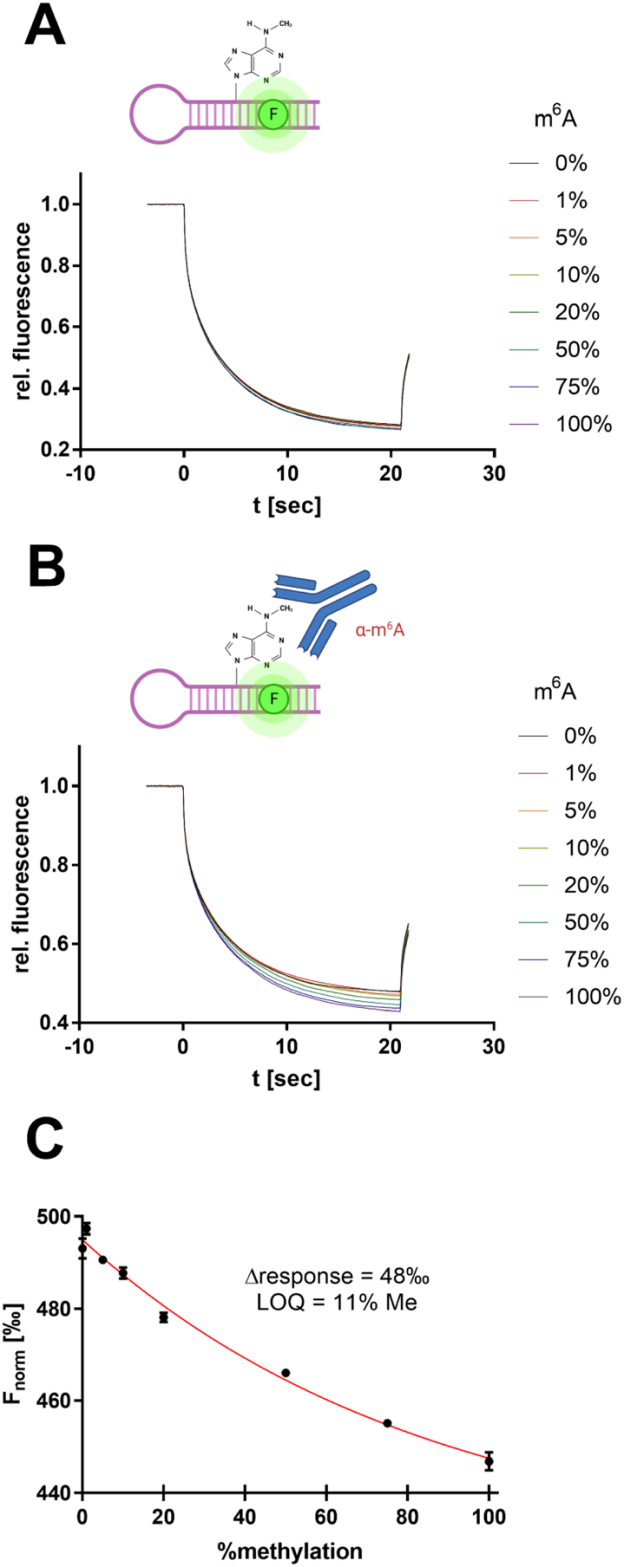
Antibody-assisted detection of RNA m^6^A modification by SybrGold MST. (A) MST traces for METTL3/14 substrate methylation levels (total RNA: 100 nM). (B) Same reaction mixture as in (A) but supplemented with 600 nM anti-m^6^A antibody clone 9B7.^40^ (C) MST response (*F*_norm_ at 10 s) as a function of substrate methylation derived from (B). The limit of quantitation (LOQ) was defined by 3*σ* around *F*_norm_ = 0%.

MST offers the potential to evaluate turnover rates of enzymatic reactions, especially in cases in which no direct detection *via* fluorescence reporter exists.^38,39^ Therefore, we sought to determine the intrinsic difference of the substrate's m^6^A modification in thermophoresis behaviour. For this purpose, we simulated different m^6^A methylation levels from 0–100% by mixing the synthetic substrate oligo with its methylated analogue at a total RNA concentration of 100 nM (Fig. 7A).

However, the use of 1× SybrGold and Syto 59 as a reporter dye resulted in very little intrinsic difference between the unmethylated and methylated oligos' *F*_norm_ values (*Δ* = 5‰), so discrimination was not sufficient for the evaluation of enzymatic turnover. Therefore, we harnessed an antibody-coupled approach to amplify the *F*_norm_ discrimination signal. The addition of an m^6^A-specific antibody (9B7 clone, 600 nM) resulted in a 10-fold dispersion (*Δ* = 48‰, LOQ = 11% methylation) of the RNA oligos' *F*_norm_ (Fig. 7B and C).^40^ This concept thus resembles an immuno-MST assay which has been previously described for non-RNA substrates but in this form claims novelty as an RNA methyltransferase assay.^41,42^

**Fig. 8:**
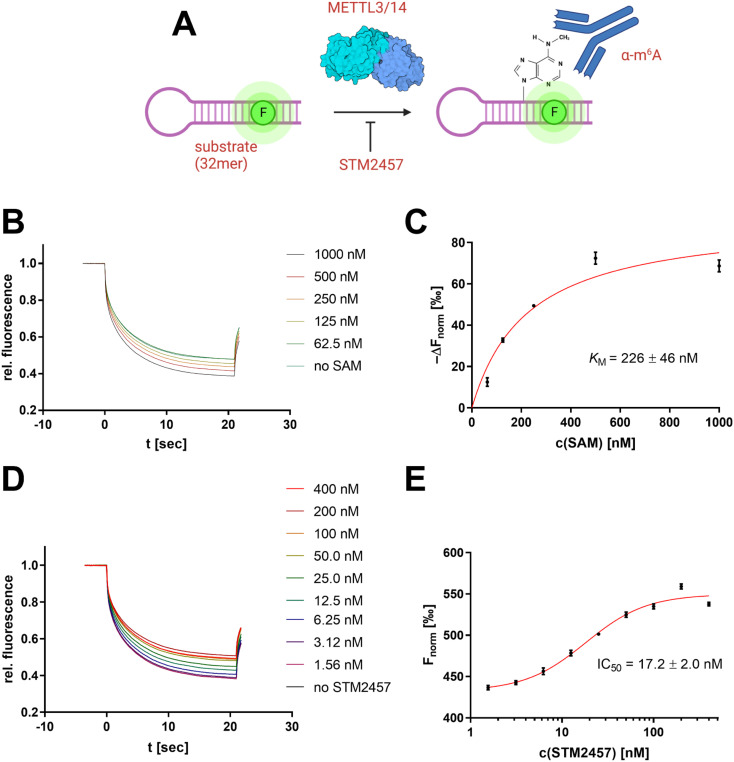
Development of an MST-based METTL3/14 assay. (A) Illustration of the assay concept: a 32mer METTL3/14 substrate oligo is converted enzymatically which can be detected using an anti-m^6^A antibody and SybrGold MST. (B) Enzymatic substrate methylation with varying concentrations of SAM as cofactor. After 2 h of incubation time, the crude reaction mixture was supplemented with the anti-m^6^A antibody and analyzed by SybrGold MST. (C) Michaelis–Menten plot of the MST response (*F*_norm_ at 10 s) *vs.* the SAM concentration. A *K*_M_ value (226 nM) was calculated by non-linear regression. (D) MST curves of the enzymatic substrate methylation in the presence of STM2457 as METTL3/14 inhibitor. (E) Determination of the STM2457 IC_50_ value by dose–response fit of *F*_norm_*vs.* [STM2457].

In this regard, the RNA substrate (120 nM), METTL3/14 enzyme (10 nM), and a variable concentration of the cofactor SAM (0–1000 nM) were incubated for 2 h at room temperature. Subsequently, the reaction was added with the anti-m^6^A antibody (600 nM) and mounted in MST capillaries as a crude reaction mixture without further purification of the RNA substrate (Fig. 8A). The resulting thermophoresis curves showed a dependence of the *F*_norm_ response on the concentration of the SAM cofactor (Fig. 8B), which can be attributed to a different methylation content of the RNA substrate. The differential analysis of this *F*_norm_ shift at different SAM concentrations agreed with the calibration of the m^6^A content (Fig. 7C) and indicated near quantitative methylation at the highest SAM concentration of 1 μM (Fig. 8C). The initial addition of SAH (10 μM) as a known inhibitor of methylation reaction yielded no shifts of the *F*_norm_ values (Fig. S7A†), confirming the effective inhibition of the enzyme and the overall assay concept. Michaelis–Menten regression of this differential methylation content (Δ*F*_norm_) allowed the determination of an apparent *K*_M_ value for SAM of 226 nM which is in agreement with a ^3^H-based enzyme assay (Fig. S7B†) and literature reports.^43^

To demonstrate the usefulness of the developed METTL3/14 assay for drug discovery applications, we also performed an inhibition experiment with the known literature inhibitor STM2457 (Fig. 8C), which revealed an apparent inhibition constant IC_50_ of 17.2 nM in agreement with the literature-determined value.^44^

## Conclusions

In conclusion, we could show that the use of non-covalent nucleic acid dyes is suitable to determine some RNA modifications by a simple “mix-and-measure” protocol. Comparing *in situ* labelled RNA samples with orthogonal ligand binding assays, we found that the usage of non-covalent dyes mostly does not alter ligand binding of the RNA molecules, and hence, this new protocol might result in a wide range of opportunities for research applications on the function of RNA molecules. Meanwhile, small modifications that do not significantly affect *F*_norm_ could be enhanced by the use of a modification-specific antibody, as shown for the METTL3/14 assay, allowing the detection of minimal chemical modifications such as single-site methylation of larger RNA molecules. We hope to have laid the first step for the development of further RNA-immuno-MST assays and for numerous studies in this field.

## Experimental section

### DNA/RNA oligos

DNA/RNA oligos except for the ones prepared by *in vitro* transcription (IVT, see below) were purchased in HPLC-purified quality (for oligo sequences and manufacturers see Tables S1 and S2†). All experiments were performed under RNase-avoiding conditions. The concentrations of RNA, DNA, and enzymes were determined by a ThermoScientific NanoDrop2000.

### RNA preparation

#### PCR

The DNA templates for the IVT were amplified using PCR. The PCR reaction mixture contained 10 nM of template DNA, 2 μM of each forward and reverse primer, 400 μM dNTP Mix (NEB), 3 mM MgCl_2_, 0.05 U μL^−1^ Q5 High-Fidelity DNA Polymerase (NEB) and 1× Q5 reaction buffer (NEB) in a final volume of 200 μL. The reaction was performed using a ^3^Prime thermal cycler with the following cycling conditions: initial denaturation (95 °C, 30 s), followed by 30 cycles of denaturation (95 °C, 30 s), annealing (temperature depends on the primer sequence and was determined by NEB T_M_ calculator, 30 s) and extension (72 °C, 45 s), and a final extension (72 °C, 2 min). The PCR product homogeneity was checked on a 0.4% agarose gel to confirm the size and purity.

#### *In vitro* transcription (IVT)

The crude PCR products were used as templates for *in vitro* transcription using the T7 polymerase system. The IVT reaction mixture contained 400 μL PCR product (without further purification), 5 mM of each NTP, 5 mM DTT, 2.5 μg mL^−1^ BSA, MgCl_2_ (concentration depends on RNA type and was determined before using micro reactions with different MgCl_2_ concentrations), 50 U mL^−1^ T7 RNA polymerase (ThermoFisher) and 1× reaction buffer (ThermoFisher) in a final volume of 1 mL. The reaction was incubated at 37 °C for 2 hours, and then, another aliquot of T7 RNA polymerase was added and incubated for 4 hours at 37 °C.

#### RNA extraction and purification

The formed pyrophosphate was removed by centrifugation at 14 000 g for 5 min directly after transcription and the cleared supernatant was transferred to a micro reaction tube for further sample processing. To remove the PCR- and IVT enzymes, a phenol/chloroform extraction was performed twice using ROTI® phenol/chloroform/isoamyl alcohol (Carl Roth) and the standard protocol from the supplier. For a better gel resolution, the extracted samples were desalted using Zeba spin desalting columns (ThermoFisher) following the standard protocol from the supplier. Furthermore, ethanol precipitation was applied to remove chloroform residues and concentrate the RNA. For this, the samples were mixed with 2.5× volume ethanol, 0.1× volume 3 M NaOAc (pH 4.7), and 1 μL glycogen (20 g L^−1^, ThermoFisher), cooled at −80 °C for 2 hours, then centrifuged for 45 min at 4 °C and 16 000 g. The pellet was washed twice with 75% ice-cold ethanol and centrifuged again for 10 min at 4 °C and 16 000 g. The air-dried pellet was redissolved in a small volume of RNase-free water and an equal volume of 2× gel loading buffer (2× TBE, 67% formamide) was added.

For the purification of RNA, a native polyacrylamide gel electrophoresis was utilized. A 10% acrylamide gel solution was prepared using the ROTIPHORESE® DNA sequencing system and 10× TBE buffer. After sample loading, the electrophoresis separation was performed at 15 W until the dye markers of the ladder showed sufficient resolution in the separation range of interest. The RNA was visualized by UV shadowing (254 nm), extracted by cutting out the bands of interest, and the excised parts were aliquoted into small pieces and frozen at −20 °C for 30 min. The frozen sample bands were extracted overnight at 15 °C in a 0.3 M NaOAc (pH 4.7) solution and the samples were centrifuged for 10 min at room temperature and 10 000 g, the supernatant and the gel residues were added into 0.45 μm Nanosep (Pall corporation) tubes and centrifuged again for 10 min and 10 000 g. The flow-through was precipitated with ethanol as described above. The pellet was redissolved in RNase-free water and stored until usage at −20 °C. The RNA product homogeneity was checked on a native 10% polyacrylamide gel to confirm the size and purity.

### Setup of a standard mix-and-measure RNA-MST experiment (non-covalent dyes)

The reaction mixtures used for a typical MST experiment contained 100 nM of the investigated RNA and 1× of the respective dye both dissolved in MST buffer (50 mM Tris–HCl pH 7.5, 100 mM KCl, and 25 mM MgCl_2_) and supplemented with ligands or additives if needed. Previous to the measurement, the RNA stock solution was heated for 5 min at 75 °C and then cooled down to room temperature in the heating block.

MST experiments were carried out using a Monolith NT.115 instrument (NanoTemper Technologies) using Monolith standard capillaries. The instrument was calibrated according to the manufacturer's instructions. The laser type was chosen dye-dependently (NanoBlue laser for SybrGold and RiboGreen and PicoRed laser for Syto dyes). General settings were applied for all MST experiments as follows: manual temperature control: 25 °C, fluorescence measurement before MST: 4 s, MST (IR laser) on: 20 s, fluorescence after MST: 2 s, delay: 25 s. The laser power was adjusted to optimize the signal-to-noise ratio and the fluorescence signal. LED and MST power settings were chosen individually for each sample by adjusting the LED power to yield fluorescence signals of at least 300 units (blue laser) or 3000 units (red laser) and the MST power to achieve an appropriate thermophoretic response (standard setting: medium MST power). MST measurements were analyzed using the NT analysis software (version 1.5.41) and exported for statistical analysis and plotting in GraphPad Prism 7.01.

### Advice for the establishment of new RNA–ligand assay systems

We recommend the following procedure for assaying new RNA targets: initially, the new experimental setup should be tested with 100 nM RNA and 1× SybrGold (for NanoBlue devices) or 1× Syto 59 (for PicoRed devices). Initial assays include four controls consisting of 100 nM RNA, 1× dye, and a known reference ligand (*c* = 10×*K*_D_) in a buffer that has been shown to be appropriate for the biological function of the RNA: (1) +RNA/+dye/+ligand, (2) +RNA/+dye/−ligand, (3) −RNA/+dye/−ligand, (4) −RNA/+dye/+ligand. Samples 3 and 4 should not show a significant thermophoresis curve due to the absence of RNA. Sample 1 should optimally result in an MST shift compared to Sample 2 as a function of reference ligand concentration.

If a ligand-dependent MST shift is not achieved with this initial setup, we recommend the following steps with the same assay setup: (1) variation of RNA dyes as described above, (2) variation of RNA concentration, (3) variation of thermophoresis settings (laser power, temperature, *etc.*).

### MST experiments with 5′-Cy5-labelled RNA

MST experiments were performed on a Monolith NT.115 using standard uncoated capillaries. The 5′-Cy5-labelled RNA was heated to 75 °C for 5 min and cooled to room temperature for 60 min. The Cy5-labelled PreQ_1_ RS and SAM-VI RS were diluted to 20 nM in MST buffer (50 mM Tris–HCl pH 7.5, 100 mM KCl, and 25 mM MgCl_2_) and supplemented with ligands in appropriate concentrations. Data acquisition was performed identically to the setup of the non-covalently labelled MST experiments (*vide supra*). MST measurements were analyzed using the NT analysis software (version 1.5.41) and exported for statistical analysis and plotting in GraphPad Prism 7.01.

### METTL3/14 MST assay (*K*_M_ & IC_50_) determination

MST-based METTL3/14 assays were carried out by mixing the RNA substrate AACUUAAUGUUGCAUUGGACUUGAGUUA (Iba, final: 120 nM), the METTL3/14 enzyme (Biomol, final: 10 nM) and a variable concentration of the cofactor *S*-adenosylmethionine (Sigma Aldrich, final: 0–1000 nM) in 50 μL of assay buffer (20 mM Tris pH 7.5, 1 mM DTT, 0.01% Triton X-100, 50 mM KCl and 0.25 mM MgCl_2_). After an incubation period of 2 h at room temperature, the reaction was quenched by the addition of SAH (Sigma Aldrich, final: 10 μM, *K*_I_ = 260 nM), added with the anti-m^6^A antibody (clone 9B7, final: 600 nM), SybrGold (final: 1×), and mounted in MST capillaries in triplicates. The laser power was adjusted to 20% for the LED laser and “high” for MST laser. MST measurements were analyzed using the NT analysis software (version 1.5.41) and exported for statistical analysis and plotting in GraphPad Prism 7.01. The decrease in *F*_norm_*vs.* [SAM] is a measure of the activity of the enzyme, and thus, the MST traces follow the Michaelis–Menten equation which was used for enzyme kinetic analysis: *v*_0_ = (*v*_max_ × [S]/*K*_M_ + [S]), where *v*_0_ is the initial reaction rate, at a given substrate concentration [S]. *v*_max_ is the maximum reaction rate, while *K*_M_ indicates [S] at which the conversion rate is half-maximal.

To determine the IC_50_ value of the reference inhibitor STM2457, the assay was performed as described above with the following adaptations. 500 nM SAM was used as substrate. STM2457 was added as DMSO stock (final DMSO conc.: 3%). The enzyme reactions were incubated for 60 min at room temperature and quenched with SAH (10 μM). *F*_norm_ values *vs.* [STM2457] were evaluated using a 4-parameter Hill equation to determine an IC_50_ value: *y*(*F*_norm_) = bottom + ([STM2457]^slope^) × (top − bottom)/([STM2457]^slope^ + IC^slope^_50_).

### METTL3/14 tritium-based assay

The radiometric METTL3/14 assay was carried out as described previously by mixing the RNA substrate AACUUAAUGUUGCAUUGGACUUGAGUUA (Iba, final: 200 nM), the METTL3/14 enzyme (Biomol, final: 20 nM) and a variable concentration of the cofactor ^3^H-SAM (Hartmann Analytics 15 Ci mmol^−1^, final: 0–1000 nM) in 10 μL of assay buffer (20 mM HEPES pH 7.5, 50 mM KCl, 250 μM MgCl_2_, 1 mM DTT, 0.01% Tween-20).^43^ The reactions were started by the addition of the cofactor ^3^H-SAM and were carried out at room temperature for 30 min. Aliquots of 8 μL were taken from the reaction mixture and spotted on Whatman glass microfiber filters (GF/C, 25 mm, Cytiva). The RNA was precipitated on the filters in 5% ice-cold TCA (Sigma Aldrich) for 35 min. The filters were washed twice with 5% TCA at room temperature for 20 and 10 min and once in EtOH for 10 min. After drying, the filters were transferred into scintillation vials and 3 mL of Gold MV liquid scintillation cocktail (PerkinElmer) was added. Scintillation was measured on a scintillation counter (TriCarb Liquid Scintillation Analyzer 4810TR) with a measurement time of 1 min. Statistical analysis and plotting were performed in GraphPad Prism 7.01 as described above.

## Data availability

The datasets supporting this article have been uploaded as part of the ESI.†

## Author contributions

Elisabeth Kallert: formal analysis, methodology, validation, writing—original draft. Malte Behrendt: methodology, validation. Ariane Frey: methodology, formal analysis. Christian Kersten: conceptualization, writing—review & editing. Fabian Barthels: conceptualization, visualization, writing—review & editing.

## Conflicts of interest

There are no conflicts to declare.

## Supplementary Material

SC-014-D3SC02993J-s001
