# Supplementary material for: Non-covalent dyes in microscale thermophoresis for studying RNA ligand interactions and modifications
Source: Chem Sci. 2023 Aug 29;14(36):9827–37. doi: 10.1039/d3sc02993j (PMC10510756; doi:10.1039/d3sc02993j)
Supplement: SC-014-D3SC02993J-s001 [file SC-014-D3SC02993J-s001.pdf]

Electronic Supplementary Material (ESI) for Chemical Science.
This journal is © The Royal Society of Chemistry 2023

Non-covalent Dyes in Microscale Thermophoresis for Studying RNA Ligand Interactions and Modifications

Elisabeth Kallert,[a] Malte Behrendt,[a] Ariane Frey,[a] Christian Kersten,[a] Fabian Barthels*[a]

[a] E. Kallert, M. Behrendt, A. Frey, Dr. C. Kersten, Dr. F. Barthels
Institute of Pharmaceutical and Biomedical Sciences
Johannes Gutenberg-University of Mainz
Staudingerweg 5, 55128 Mainz, Germany
E-mail: barthels@uni-mainz.de

RNA and DNA oligo sequences

Table S1. Oligos used for RNA-MST experiments. **Category 1**: no structure, <10mer; **category 2**: no structure, 10–15mer; **category 3**: no structure, 16–20mer; **category 4**: hairpin, 16–20mer; **category 5**: bulge/internal loop + hairpin, 21–25mer; **category 6**: internal loop + hairpin, 26–30mer; **category 7**: bulge + internal loop + hairpin(s), 31–50mer; **category 8**: 2 internal loops + 1 hairpin, 50mer; **category 9**: 3 internal loops + 1 hairpin, 60mer; **category 10**: various functional RNA.

OligoID	Cat.	RNA sequence	Manufacturer
**MH993**	1	GUUAUUC	Iba
**MH996**	1	UUUUAUUUU	Biomers
**MH474**	1	GGUACCCAA	Iba
**MH122**	2	ACAGCUAUCCAUUG	Iba
**MH847**	2	UCUGAGGGUCCAGGG	Biomers
**MH268**	2	CCCGGUAAUCGCAUA	Salifu
**MH266**	3	AAACUUAAAACUUUACUGU	Salifu
**MH907**	3	ACAAGAAGGGUUUGAUCAUC	Iba
**MH272**	3	AGAAAUAUGUCUGAUAAA	Salifu
**MH355**	4	CGCGGGAGACCGGGGUUCGA	Dharmacon
**MH261**	4	GCUUGAAACCAGCUUUGGG	Salifu
**MH1428**	4	GGAUCCUCUAGAGUCGACCU	Idt
**MH515**	5	GGUGGGGUUCCCGAGCGGCCAAAG	Iba
**MH499**	5	GUUCGAUCCACAGAAUUCGCACCA	Biomers
**MH649**	5	GGUUCGAUUCCCCGACGGGGAGCCA	Iba
**MH857**	6	UCUAGAGGAUCCCGGGUACCGAGCUC	Biomers
**MH876**	6	UGCCGCCCGUCUUGAAACACGGACCAAGGA	Iba
**MH1145**	6	AACUUAAUGUUUUUGCAUUGGACUUUGAGUUA	Iba
**MH761**	7	GGCGUGUAGGAUAUUAAAUCAUACCAGAUCGCGG	Iba
**MH646**	7	UCCUCGUUAGUAUAGUGGUGAGUAUCCCCGCCUAUAUC	Iba
**MH566**	7	GUCACGCGGGAGACCGGGGUUCGAUUCCCCGACGGGGAGCCA	Iba
**MH723**	8	CCGCGAUCUGGAGAGGUGAAGAAUACGACCACCUACAAGAAGGACACGCC	Iba
**MH721**	8	CCGCGAUCUGGAGAGGUGAAGAAUACGACCACCUAAGAAGGACACGCC	Iba
**MH762**	8	CCGCGAUCUGGAGAGGUGAAGAAUUCGACCACCUACCAGAAGGACACGCC	Iba
**MH950**	9	CGGAGCCAGCGAGUCUAACCUUGGCCGAGAGGUCUUGGUAAUCUUGUGAAACUC CGUCGU	Iba

**MH952**	9	GGCCUCAGGAUCUGCUUAGAGAAGGGGGCAACUCCAUCUCAGAGCGGAGAAUUUGGACAA	Iba
**MH953**	9	UCUUGGAUUUAUUGAAGACUAACUACUGCGAAAGCAUUUGCCAAGGACGUUUUCAUUAAU	Iba
**tRNA^{Asp}**	10	UCCUCGUUAGUAUAGUGGUGAGUAUCCCCGCCUGUCACGCGGGAGACCGGGGUUCGAUUCCCCGACGGGGAG	In house IVT
**PreQ$_1$ RS** (*Thermoanaerobacter tengcongensis*)	10	GGAGAGGUUCUAGCUACACCUCUAUAAAAAACUAA	biomers
**SAM-I RS** (*Thermoanaerobacter tengcongensis*)	10	GGCUUAUCAAGAGAGGGGGAGCGACUGGCGCGAAGAACCCCGGCAACCAGAAAUGGUGCCAAUUCCUGCAGCGGAAACGUUGAAAGAUGAGCCG	In house IVT
**SAM-VI RS** (*Bifidobacterium angulatum*)	–	GGCAUUGUGCCUCGCAUUGCACUCCGCGGGGCGAUAAGUCCUGAAAAGGGAUGUC	In house IVT
**Cy5-PreQ$_1$ RS** (*Bacillus subtilis*)	–	Cy5-AGAGGUUCUAGCUACACCCUCUAUAAAAAACUAA	Eurofins Genomics
**Cy5-SAM-VI RS** (*Bifidobacterium angulatum*)	–	Cy5-GGCAUUGUGCCUCGCAUUGCACUCCGCGGGGCGAUAAGUCCUGAAAAGGGAUGUC	Eurofins Genomics
**AdoCbl riboswitch** (Marine metagenome *env8* 4WJvar)	–	GGCCUGAAAGCGUGGUGGGGAAGUGACGUGAAAUUCGUCCAGAUUACUUGAUACGGUUAUACUCCGAGUGCCACCCAGGCCAUACAACGAGCAAGGAGACUC	GenScript
**Fluoride riboswitch** (*Thermotoga petrophila*)	–	GGGCGAUGAGGCCCGCCCAAACUGCCCUGAAAAGGGCUGAUGGCCUCUACUG	GenScript
**Neomycin aptamer** (synthetic)	–	GGCUGCUUGUCCUUUAAUGGUCCAGUC	GenScript
**METTL3/14 substrate**	–	AACUUAAUGUUGCAUUGGACUUGAGUUA	Iba

Table S2. DNA templates and primers used for PCR and IVT.

OligoID	description	DNA sequence	Manufacturer
**MH53**	Universal forward primer	CGCGCGAAGCTTAATACGACTCACTATA	IDT
**EK0004**	Reverse primer SAM-I RS	CGGCTCATCTTTCAACGTTTCCG	IDT
**EK0010**	Reverse primer SAM-VI RS	GACATCCCTTTTCAGGACTTATCGCCCCG	IDT
**MH1102**	Reverse primer $tRNA^{Asp}$	TGGCGGGCCGTCG	Iba
**EK0003**	Template SAM-I RS	CGGCTCATCTTTCAACGTTTCCGCTGCAGGAATTGGCACCATTTCTGGTTGCCGGGGTTCTTC<br>GCGCCAGTCGCTCCCCCTCTCTTGATAAGCCTATAGTGAGTCGTATTA	IDT
**EK0009**	Template SAM-VI RS	GACATCCCTTTTCAGGACTTATCGCCCCGCGGAGTGCAATGCGAGGCACAATGCCTATAGT<br>GAGTCGTATTA	IDT
**MH1101**	Template $tRNA^{Asp}$	TGGCGGGCCGTCGGGGAATCGAACCCCGGTCTCCCGCGTGACAGGCGGGGATACTCACCA<br>CTATACTAACGACCCTATAGTGAGTCGTATT	Iba

Supplementary Figures

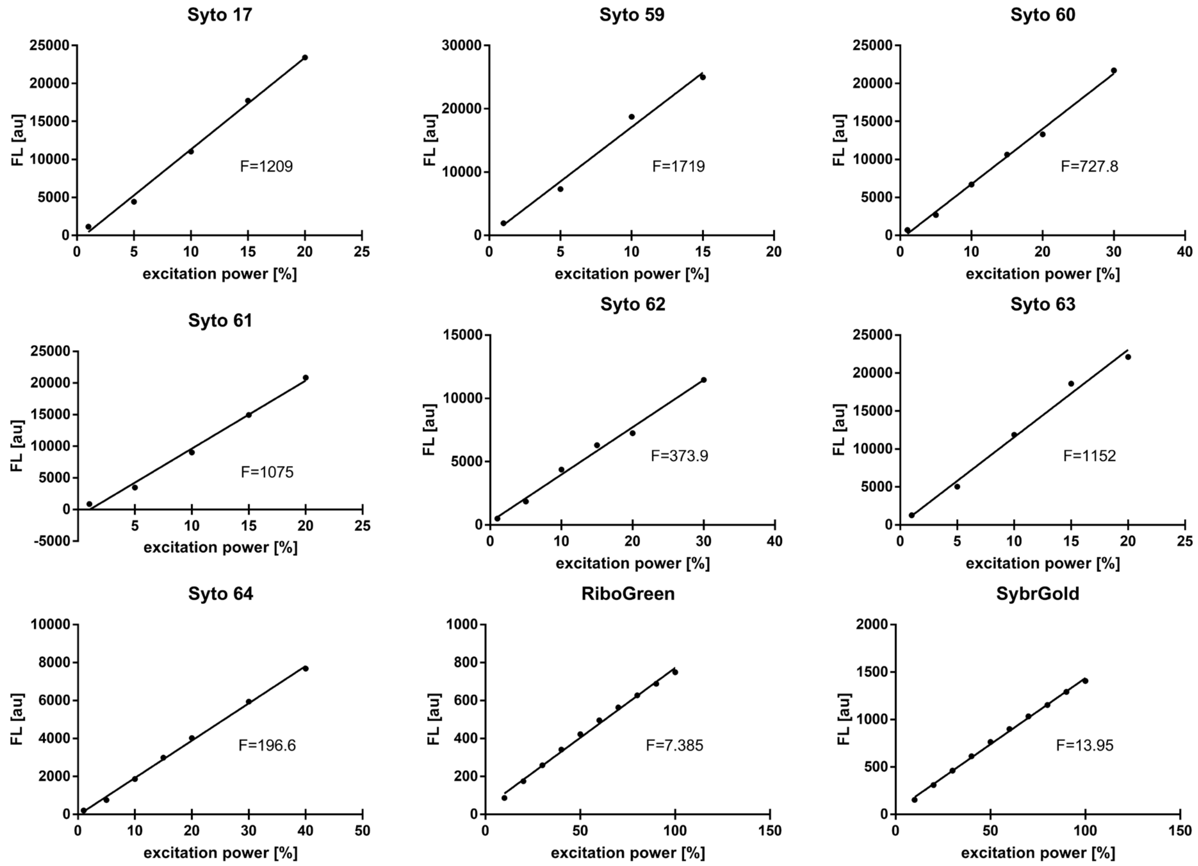

Figure S1. Regression plots for dye fluorescence normalization. Each dye (1×) was mixed with 100 nM of MH646 as RNA model oligonucleotide (UCCUCGUUAGUAUAGUGGUGAGUAUCCCCGCCUAUAUC). The initial fluorescence intensity as a function of the excitation power was determined pre-MST with either the NanoBlue laser for SybrGold and RiboGreen or the PicoRed laser for the Syto dyes. Linear regression yielded a slope-derived sensitivity factor (F) for the normalization of fluorescence intensity during structure-activity-relationship experiments.

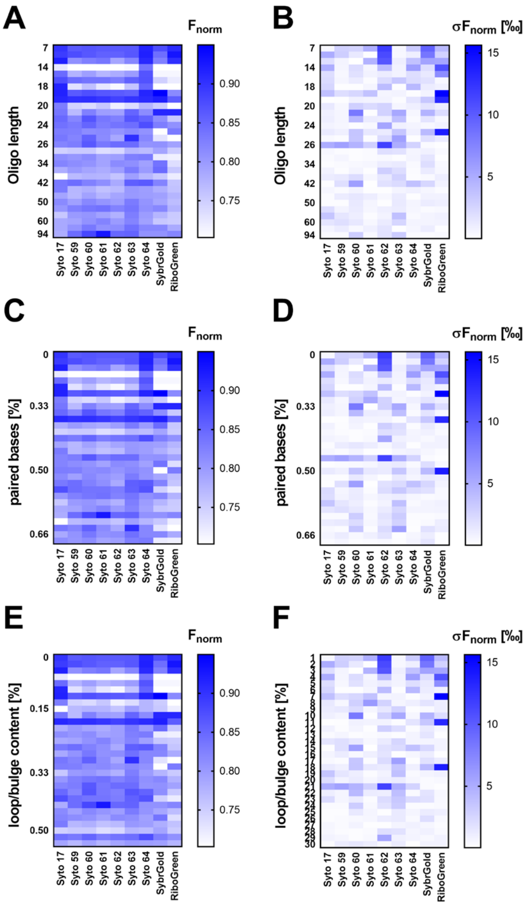

Figure S2. Structural RNA-dependent effects on F_{norm}. (**A**) Correlation between oligo length and F_{norm}. (**B**) Correlation between oligo length and σF_{norm}. (**C**) Correlation between hybridized base content and F_{norm}. (**D**) Correlation between hybridized base content and σF_{norm}. (**E**) Correlation between loop/bulge content and F_{norm}. (**F**) Correlation between loop/bulge content and σF_{norm}.

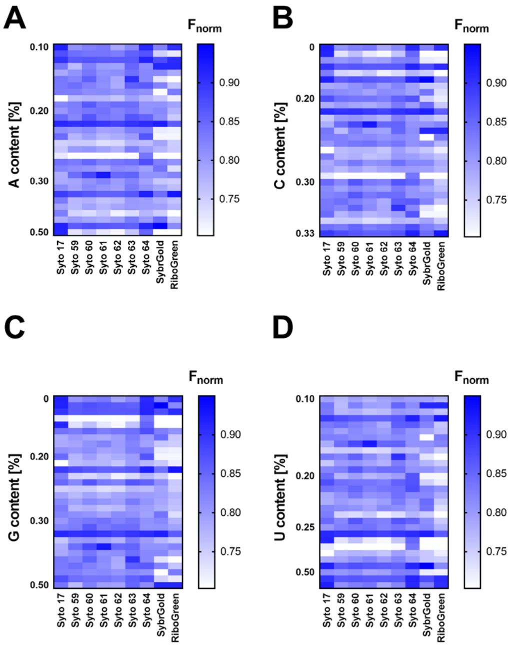

Figure S3. RNA sequence-dependent effects on F_{norm}. (**A**) Correlation between adenine content and F_{norm}. (**B**) Correlation between cytosine content and F_{norm}. (**C**) Correlation between guanine content and F_{norm}. (**D**) Correlation between uridine content and F_{norm}.

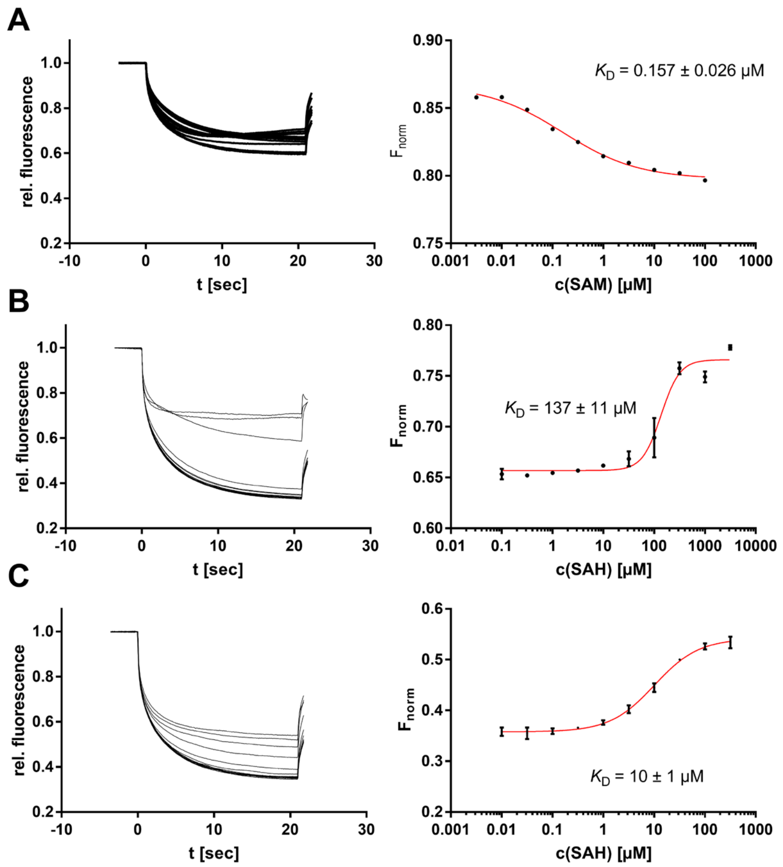

Figure S4. Analysis of RNA-ligand interaction by in situ labelling with SybrGold (1×). (**A**) Interaction between SAM-I RS and SAM (F_{norm} at 1 s). (**B**) SAM-I RS and SAH (F_{norm} at 1 s). (**C**) SAM-VI RS and SAH (F_{norm} at 15 s).

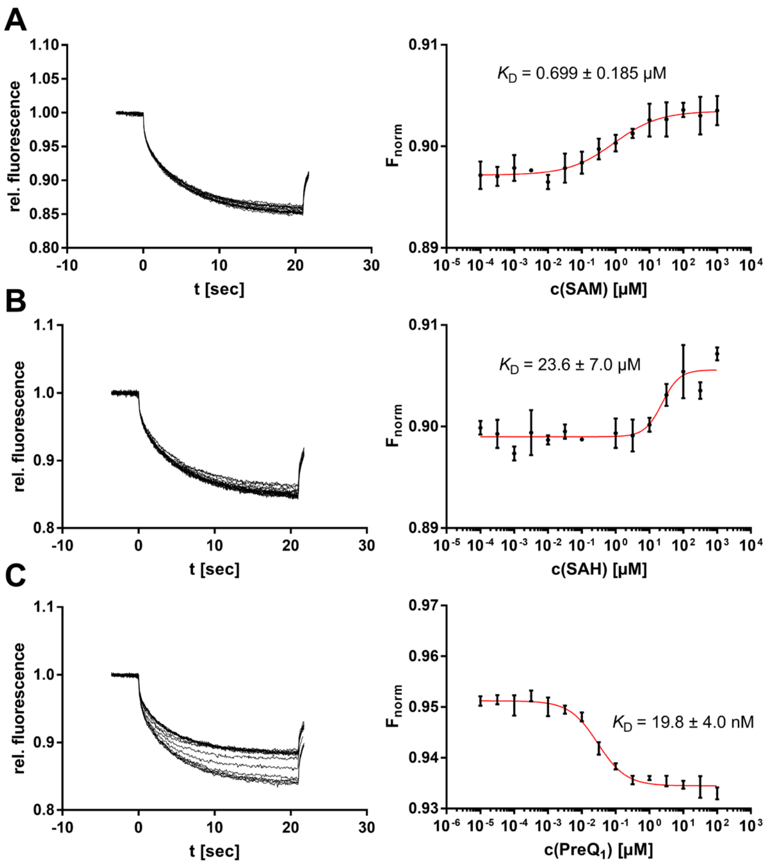

Figure S5. Analysis of RNA-ligand interaction by Cy5-labelled RNA-MST (**A**) Interaction between SAM-VI RS and SAM (F_{norm} at 5 s). (**B**) SAM-VI RS and SAH (F_{norm} at 5 s). (**C**) $PreQ_1$ RS and $PreQ_1$ (F_{norm} at 1 s).

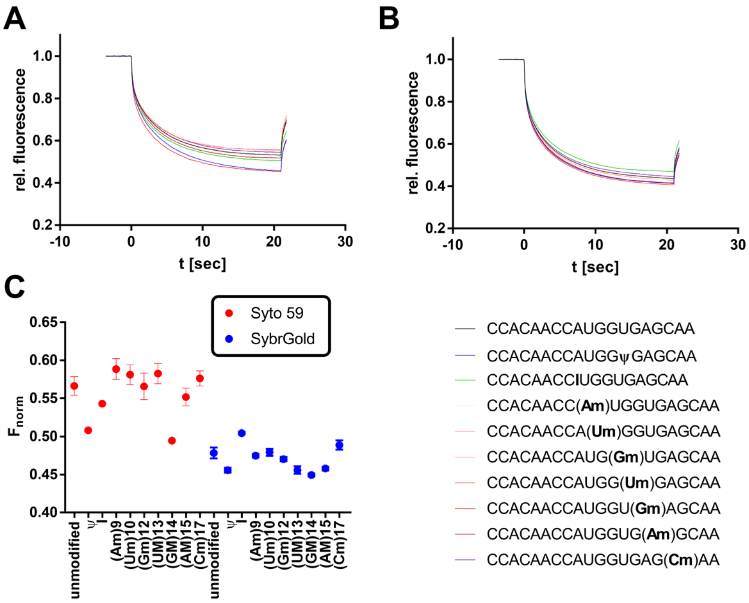

Figure S6. Analysis of various modifications of the model oligo CCACAACCAUGGUGAGCAA (100 nM) by MST. (**A**) Thermophoresis curves for the usage of Syto 59 as a reporter dye. (**B**) Thermophoresis curves for the usage of SybrGold as a reporter dye. (**C**) MST response (F_{norm} at 10 s) of the modified oligos in comparison to their unmodified analogues.

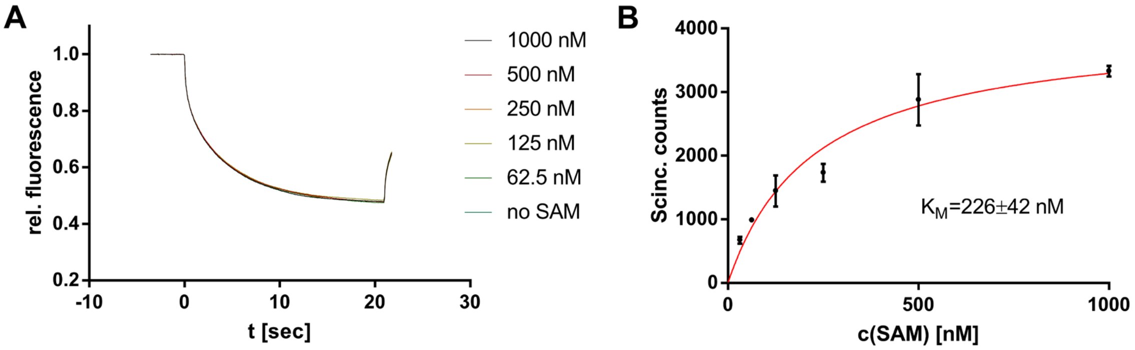

Figure S7. Supplementary data for the establishment of the METTL3/14 assay. (**A**) Thermophoresis curves for the METTL3/14 reaction inhibited by the initial addition of SAH (10 µM). Enzymatic substrate methylation with varying concentrations of SAM as the cofactor. After 2 h of incubation time, the crude reaction mixture was supplemented with the anti-m^{6}A antibody and analyzed by SybrGold MST. (**B**) Tritium-based METTL3/14 methyltransferase assay. The Michaelis-Menten plot was derived from radiometric analysis of ^{3}H incorporation into RNA substrate AACUUAAUGUUUGCAUUGGACUUGAGUUA. A K_M value (226 nM) was calculated by non-linear regression (Michaelis Menten).

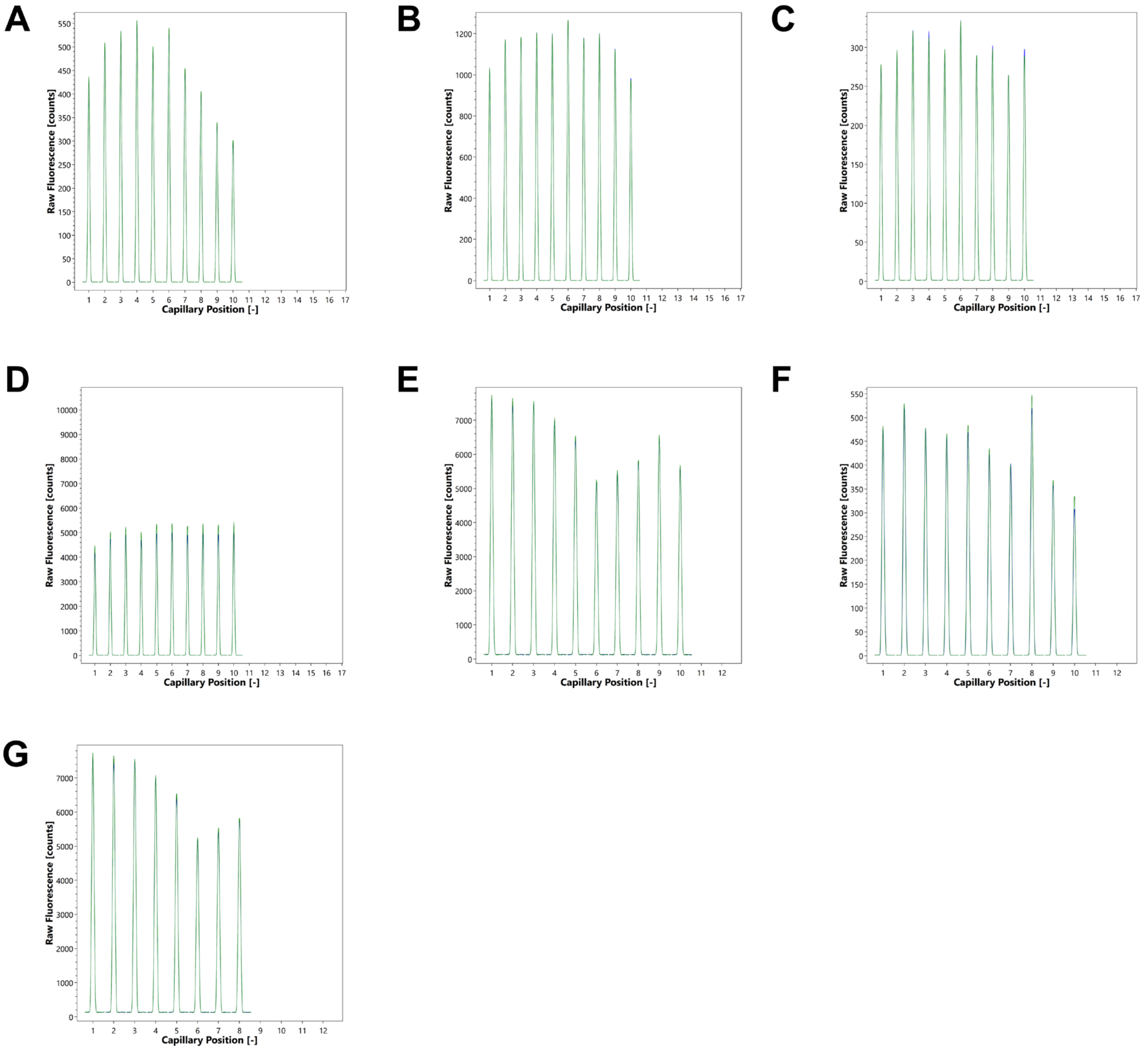

Figure S8. Capillary scans of RNA-ligand interaction samples for the assessment of dye displacement by ligand binding. Capillary scans are presented with increasing concentration corresponding to the MST curves in Figure 4 and Figure S4. (**A**) $PreQ_1$ RS vs. $PreQ_1$. (**B**) SAM-I RS vs. SAM. (**C**) SAM-VI RS vs. SAM. (**D**) Fluoride RS vs. potassium fluoride. (**E**) AdoCbl RS vs. adenosyl cobalamin. (**F**) DNMT2 vs. tRNA. (**G**) Neomycin aptamer vs. neomycin.
